# Complete Genome Sequence of a Brazil-Type *Avian coronavirus* Detected in a Chicken

**DOI:** 10.1128/genomeA.01135-16

**Published:** 2016-10-13

**Authors:** Paulo E. Brandão, Giselle R. R. Ayres, Carolina A. Torres, Laura Y. B. Villarreal, Aline S. Hora, Sueli A. Taniwaki

**Affiliations:** a Department of Preventive Veterinary Medicine and Animal Health, School of Veterinary Medicine, University of São Paulo, São Paulo, Brazil; b MSD Animal Health, São Paulo, Brazil

## Abstract

*Avian coronavirus* is the causative agent of infectious bronchitis in chickens, leading to multisystemic disease that might be controlled if adequate vaccine strains are used. This paper reports the first complete genome sequence of a Brazil type of this virus (27,615 nucleotides [nt]) isolated from the kidneys of a chicken.

## GENOME ANNOUNCEMENT

*Avian coronavirus* (AvCoV) (*Nidovirales*:*Coronaviridae*:*Coronavirinae*:*Gammacoronavirus*) host-type avian infectious bronchitis virus (IBV) in chickens occurs as multiple types grouped in six genotypes, with a total of 32 lineages based on spike S gene comparisons, and it is involved in multisystem highly contagious infections of chickens (1, 2). The positive-sense, single-stranded, 5′-capped genomic RNA (ca. 27 kb) codes for the replicase complex (open reading frame 1 [ORF1]) and five structural proteins on the remaining 1/3 at the 3′ end: spike S, envelope protein E, membrane protein N, and nucleocapsid protein N, besides the accessory proteins 3a, 3b, 5a, and 5b, with an untranslated region (UTR) at both the 5′ and 3′ ends (3, 4). Although a typical Brazilian type of IBV has been reported (5), no complete genome sequence was available hitherto for this type.

The Gammacoronavirus/AvCoV/chicken/Brazil/23/2013 IBV strain was isolated after three serial passages in 11-day-old chicken embryonated eggs from the kidneys of a chicken from Brazil collected in 2013. Random double-stranded cDNAs were obtained from clarified (12,000 × g/15 min/4°C), filtered (0.45-µm-pore-size), and DNase/RNase-treated allantoic fluid using SuperScript III and Klenow exo-DNA polymerase (Life Technologies) after total RNA extraction with TRIzol reagent (Life Technologies) and RNeasy minikit (Qiagen). Libraries and sequencing kits were by Illumina (Nextera XT index, Nextera XT DNA), and reads were obtained with a NextSeq 500 (Illumina) using the NextSeq500 Mid output version 2 kit (150 cycles). The consensus sequence was assembled with CLC Genomics Workbench version 8.5.1 (Qiagen), using AvCoV sequence with accession number KJ425485.1 as a reference and with reads that were trimmed for quality (limit, 0.05). A total of 10,274,194 paired-end reads were obtained, and from this, 7,558,298 reads were mapped.

The resulting genome was 27,615 nucleotides (nt) long, including the poly(A) tail, and is organized as 5′ UTR (nt 1 to 528), ORF1a (nt 529 to 12318), and ORF1ab (nt 12318 to 20357), with a ribosomal frameshift between these two ORFs, followed by the genes spike S (nt 20308 to 23817), 3a (nt 23817 to 23990), 3b (23990 to 24181), envelope E (nt 24165 to 24488), membrane M (nt 24457 to 25137), 5a (nt 25497 to 25694), 5b (nt 25691 to 25939) and nucleocapsid N (25882 to 27111), with a 3′ UTR at nt 27112 to 27615, including the poly(A) tail.

Spike sequence-based analysis revealed that strain Gammacoronavirus/AvCoV/chicken/Brazil/23/2013 belongs to the GI-11 AvCoV lineage (Brazil type) with only 82.9% nt identity with the Massachusetts (GI-1) type used as a live vaccine in Brazil against avian infectious bronchitis; this low identity is one of the reasons for the lower protection given by this vaccine reported after challenges with the Brazil type (6). This full genome of a Brazil type IBV will now allow for improved analyses on the origins of this lineage and a better association with vaccine protection studies.

### Accession number(s).

The Gammacoronavirus/AvCoV/chicken/Brazil/23/2013 complete genome sequence is deposited in GenBank under the accession number KX258195.
